# Physiological Functions of the By-Products of Passion Fruit: Processing, Characteristics and Their Applications in Food Product Development

**DOI:** 10.3390/foods14091643

**Published:** 2025-05-07

**Authors:** Zhaohan Liu, Xiaonan Wang, Qianwen Li, Xiaojing Kang, Yan Li, Chunmiao Gong, Yang Liu, Han Chen

**Affiliations:** 1 Food Laboratory of Zhongyuan, Luohe 462333, China; liuzhaohan186@163.com (Z.L.); wangxiaonan@zyfoodlab.com (X.W.); liqianwen202206@163.com (Q.L.); papumoon@163.com (Y.L.); gongchunmiao@gmail.com (C.G.); 15510360925@163.com (Y.L.); 2 FLZ-Sunjock Dairy Co. Joint Laboratory, Luohe 462000, China; tn2020036@zyfoodlab.com; 3 Food Laboratory of Zhongyuan, China Agricultural University, Beijing 100083, China

**Keywords:** passion fruit, dietary fiber, polyphenols, functional properties, intestinal flora, foods

## Abstract

The by-products of passion fruit are typically discarded during processing, contributing to resource waste and environmental harm. These residues are rich in dietary fiber and polyphenols, compounds linked to health benefits, including blood sugar regulation, improved lipid profiles, gut microbiome balance, and weight management. Beyond their nutritional value, these by-products possess dual functional roles in food systems: their bioactive components act as natural fortifiers and health-promoting agents. Recent studies indicate they can enhance food quality by improving water retention and texture while serving as prebiotics to promote beneficial gut bacteria growth. This dual functionality supports both food innovation and metabolic health, particularly in reducing post-meal blood sugar spikes. To advance research and industry applications, this review synthesizes recent findings on the nutritional properties of passion fruit by-products and their use in food products such as dairy, pasta, and meat. The analysis aims to guide the sustainable utilization of these underrated resources and expand their role in functional food development.

## 1. Introduction

Passion fruit (Passiflora edulis Sims), a perennial evergreen vine of the *Passiflora genus* [1], emits a distinctive aromatic profile upon ripening, characterized by complex notes of pomegranate, pineapple, strawberry, and lemon. This unique olfactory characteristic has established its prominence in the production of juices, fruit wines, vinegar, composite beverages, and probiotic formulations, thereby demonstrating its substantial utility within the food sector [2]. The rapid expansion of passion fruit processing has concurrently generated considerable quantities of by-products, presenting both economic and environmental concerns. Among these, passion fruit peel (PFP) and passion fruit seeds (PFS) are of particular significance. PFP constitutes 50–55% of the total fruit mass, whereas PFS accounts for approximately 4–12%. Both by-products are nutritionally valuable, containing abundant bioactive constituents such as dietary fiber (DF), polyphenols, and plant-derived pigments [3,4]. Notably, Leão et al. (2014) [5] demonstrated that oil extracted from PFP exhibits an aromatic intensity two to threefold greater than that of the fruit pulp. Chromatographic analysis revealed the presence of 49 volatile compounds, including ethyl butyrate, ethyl hexanoate, and ethyl acetate, indicating the substantial potential for flavor applications. Furthermore, Oliveira et al. (2016) [3] reported that defatted PFS retains significant phenolic content and demonstrates antimicrobial activity against *Escherichia coli* and *Listeria monocytogenes*.

In contemporary diets, the increasing prevalence of refined food products has led to a marked reduction in the consumption of coarse grains, which represent a key dietary source of DF. This dietary shift has contributed to nutritional imbalances and has been epidemiologically linked to the rising incidence of modern metabolic disorders, including obesity, diabetes, gastrointestinal dysfunction, and cardiovascular diseases [6]. Polyphenols, a class of naturally occurring antioxidants ubiquitous in plant matrices, play a crucial role in mitigating oxidative cellular damage. Their capacity to inhibit low-density lipoprotein (LDL) oxidation contributes to attenuated inflammatory responses and confers cardioprotective effects [7]. Notably, PFBs are particularly abundant in both DF and polyphenolic compounds, positioning them as functional ingredients with potential preventive applications against these conditions [8]. Therefore, incorporating PFBs into food development can create new products with higher nutritional value or unique physiological benefits. This approach also has the potential to reduce the environmental impact of large quantities of industrial by-products and increase the added value of PFBs.

Despite the remarkable health benefits and industrial potential of PFBs, a systematic review of their functional properties and food applications remains lacking. Current research predominantly focuses on the extraction and identification of individual bioactive components (such as DF or polyphenols), while significant knowledge gaps persist regarding their synergistic mechanisms, stability in practical food applications, and validated health effects [6,9]. In contrast to the existing literature, this work systematically consolidates the functional characteristics of PFB, with particular emphasis on their lipid-lowering, hypoglycemic, gut microbiota-modulating, and weight management properties. Furthermore, we comprehensively evaluate current applications of PFB in the food industry, including their incorporation in dairy products, pasta, and meat products. By synthesizing current research and cutting-edge developments, this review proposes future research directions to facilitate the valorization of PFB. Our findings provide a scientific foundation for their utilization in nutraceutical applications and promote industrial adoption in the nutrition and health sectors.

## 2. Main Components of PFB

PFBs represent a nutritionally dense matrix containing substantial quantities of bioactive compounds, rendering them highly valuable for functional food applications. Of particular significance is the soluble dietary fiber (SDF) content in PFP-DF, which reaches 20.51% and demonstrates considerable physiological benefits [4]. These by-products are notably rich in polyphenolic compounds, including quercetin and kaempferol, which exhibit pronounced antioxidant capacity. Additionally, PFBs contain a diverse array of nutritionally relevant components, such as plant-derived pigments and unsaturated fatty acids. This unique compositional profile suggests extensive potential for utilization across multiple food industry applications [10,11].

### 2.1. DF

DF is formally defined as carbohydrate polymers comprising ten or more monomeric units that resist hydrolysis by endogenous enzymes in the human small intestine. DF includes intrinsic carbohydrate polymers naturally present in food matrices, extracted or isolated polymers from edible raw materials with substantiated physiological benefits, and synthetic carbohydrate polymers demonstrating analogous health-promoting properties [12]. Research indicates that the physiological effects of DF are closely associated with the ratio of SDF to insoluble dietary fiber (IDF) [13]. This correlation stems from the distinct mechanisms of action of SDF and IDF: IDF primarily increases fecal volume through water retention, whereas SDF is readily fermented by microorganisms in the colon, thereby effectively promoting the proliferation of beneficial gut microbiota. Notably, when the SDF content exceeds 10%, its physiological activity is significantly enhanced, yielding pronounced health benefits. However, the SDF content in most DF sources is less than 3%, resulting in relatively limited physiological activity [14]. In this regard, PFB demonstrates unique advantages. Studies have shown that PFP contains a remarkably high total dietary fiber (TDF) content of over 78%, of which SDF accounts for 20.51% [4], significantly exceeding that of conventional DF sources. Furthermore, the SDF content in PFS can reach 5.12% [15], a characteristic that renders it a high-quality DF source with considerable development potential.

**Figure 1:**
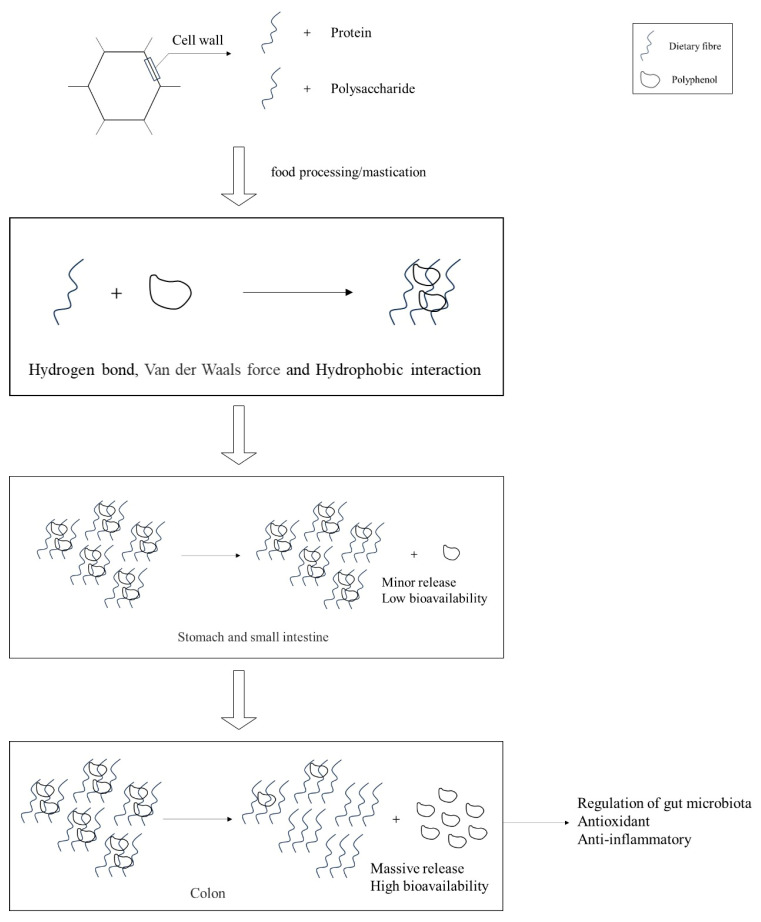
Interaction and liberation processes of DF and polyphenols.

The compositional analysis of DF and bioactive constituents in PFB is summarized in Table 1. These functional components can be isolated through various extraction methodologies, including chemical, physical, and enzymatic approaches. Notably, DF derived from PFB exhibits a distinctive compositional profile, being particularly rich in high-quality pectin compared with conventional DF sources. The binding and release processes of polyphenols with DF are illustrated in Figure 1. The high phenolic content in fruit peels can interact with polysaccharides and proteins in the cell wall via hydrophobic aromatic rings and hydrophilic hydroxyl groups [16]. When cellular structures are disrupted during processing or mastication, phenolic compounds typically bind to DF through non-covalent interactions, including hydrogen bonding, van der Waals forces, and hydrophobic effects. In this process, DF not only immobilizes phenolic compounds via intermolecular forces but also encapsulates them within a complex macromolecular matrix, forming stable complexes. During the initial stages of gastrointestinal digestion (gastric and small intestinal phases), this bound state significantly reduces the bioavailability of phenolic compounds, causing them to persist primarily as bound polyphenols. However, upon reaching the colonic phase, the complexes are gradually degraded by gut microbiota and specific enzymes, releasing free polyphenols [17]. These liberated polyphenols are subsequently absorbed in the colon, where they exert various bioactive effects, including antioxidant and anti-inflammatory activities, as well as modulation of the gut microbiota [18].

### 2.2. Polyphenol

Polyphenols, characterized by at least two phenyl rings and one or more hydroxyl groups, exhibit structural versatility that enables interactions with carbohydrate residues such as monosaccharides and polysaccharides [27]. Their biological properties and antioxidant activities are intrinsically linked to their chemical structure, leading to significant variability in bioavailability and functionality across different sources [28].

In plant-based foods, polyphenols are categorized into flavonoids (including flavanones, flavanols, isoflavones, and flavones) and phenolic acids (such as hydrocinnamic acids and hydroxybenzoic acids) [7]. The phenolic profile of PFP includes flavanols (such as kaempferol), flavones (such as quercetin), and anthocyanins [10]. These active components offer numerous physiological benefits, such as antioxidant properties, promoting fat combustion, and regulating blood sugar levels [25]. However, research on polyphenols in PFB remains limited, with existing studies often employing disparate extraction methodologies that complicate the direct comparison of results. Studies on PFB polyphenols reveal notable variability in compound concentrations, potentially influenced by cultivar differences, cultivation conditions, or extraction techniques. Studies indicate that orange varieties exhibit higher total phenolic content, whereas purple cultivars demonstrate significantly greater anthocyanin levels in both peels and seeds compared with their yellow and orange counterparts. In contrast, although yellow varieties contain relatively lower phenolic content, they are notably richer in DF [19]. Nevertheless, comparative studies on the bioactive composition of PFB from different geographical origins remain scarce, highlighting an urgent need for more systematic investigations in this field.

Novel techniques such as pressurized liquid extraction (PLE), ultrasound-assisted extraction (UAE), and microwave-assisted extraction (MAE) have demonstrated considerable potential in the field of polyphenol extraction. For instance, Montenegro et al. (2021) [29] reported that PLE and MAE yielded polyphenol contents of 3000 ± 70 mg GAE/kg and 2000 ± 130 mg GAE/kg, respectively, from spinach by-products, whereas UAE produced a lower yield of 400 ± 100 mg GAE/kg. Oliveira et al. (2022) [30] compared the efficacy of UAE and PLE for polyphenol extraction from Citrus latifólia Tan. The total phenolic content obtained via UAE varied with power, extraction time, and solid-to-liquid ratio, ranging from 1.85 to 2.33 mg GAE/g, accompanied by an oxygen radical absorbance capacity (ORAC) of 27.42–46.42 mg antioxidant activity of PLE were highly dependent on temperature and duration, peaking at 110 °C and 40 min (17.66 ± 0.04 mg GAE/g, ORAC 90.11 ± 0.76 mg Trolox Equivalent (TE)/g). These studies confirm that both emerging technologies significantly enhance TPC and antioxidant levels compared with conventional methods. Although PLE requires prolonged extraction times, it achieves superior polyphenol yields. Nevertheless, the absence of standardized protocols has led to ongoing debate regarding optimal extraction conditions and maximal recovery of bioactive compounds [22,30].

### 2.3. Other

PFB is rich in bioactive compounds, including plant pigments and unsaturated fatty acids [31].

PFP is a natural source of vibrant colors and an excellent provider of natural pigments. Ghada et al. (2020) [32] conducted a phytochemical investigation of PFP, demonstrating their viability as a sustainable botanical source of anthocyanins. Research revealed that these pigmented compounds exhibit a multi-mechanistic bioactivity profile, including extracted pigments from PFP that were not found in PFS. These include anthocyanins, such as cyanidin-3-glucoside (9.8 μg/10 mg), cyanidin-3-rutinoside (1.1 μg/10 mg), peonidin-3-glucoside (0.8 μg/10 mg), and callistephin (0.2 μg/10 mg) [31]. These thermally stable pigments represent an economical natural alternative to synthetic red food colorants. Moreover, PFS has a high content of unsaturated fatty acids. Chen et al. (2025) [11] employed a low-temperature continuous phase extraction methodology to obtain PFS oil, achieving a notable extraction efficiency of 20.37% (*w*/*w*). Analytical characterization revealed a nutrient-dense profile comprising 84.00% unsaturated fatty acids, 21.03 μg/g vitamin E (α-tocopherol equivalent), and 6.28 μg/g β-carotene. Notably, the oil demonstrated potent hypolipidemic activity, exhibiting IC50 values of 97.19 μg/mL and 48.08 μg/mL against pancreatic lipase and cholesterol esterase, respectively. These findings align with earlier work by Malacrida et al. (2012) [33], who reported an 87.59% total unsaturated fatty acid composition in PFS oil, comprising linoleic (73.14%) and oleic acids (13.83%). The oil was further characterized by substantial tocopherol content (499.30 mg/kg) and phenolic constituents (1314.13 mg GAE/kg), confirming its dual functionality as both a lipid source and phytochemical reservoir. Cavalcanti et al. (2024) [34] provided detailed fatty acid profiling through gas chromatography, identifying linoleic acid (Omega-6) as the predominant constituent (68.8%), followed by Omega-9 (16.1%), with palmitic acid representing the primary saturated component (10.61%). Collectively, these studies substantiate the nutritional merit of PFS oil as a premium plant-derived lipid source.

## 3. Functional Characteristics of PFB

PFB demonstrates multiple physiological benefits with significant health implications. Scientific evidence indicates that PFB exerts hypolipidemic effects by inhibiting cholesterol absorption, stimulating bile acid secretion, and modulating key blood lipid parameters. In glycemic control, PFB has been shown to reduce carbohydrate bioavailability while improving insulin sensitivity through multiple pathways. Furthermore, PFB exhibits prebiotic properties by selectively promoting beneficial gut microbiota, suppressing pathogenic bacterial growth, and enhancing intestinal barrier function. With regard to weight management, PFB demonstrates anti-obesity potential through mechanisms including the inhibition of adipogenesis, attenuation of low-grade inflammation, and reduction in adipose tissue deposition. As summarized in Table 2, these scientifically validated health benefits position PFB as a promising functional ingredient for nutraceutical applications and health-focused food product development.

### 3.1. Lowers Blood Lipids

Elevated plasma cholesterol levels are a well-known independent risk factor for cardiovascular diseases. High blood cholesterol during childhood can increase the risk of developing atherosclerosis and coronary heart disease later in life [50]. Extensive research has explored the mechanisms by which DF can lower blood lipid levels. Specifically, its intake has been shown to reduce serum LDL-cholesterol levels, decreasing cardiovascular disease risk [51]. Furthermore, studies indicate that consuming approximately 6 g of SDF daily can lower serum LDL-cholesterol levels by about 5.4% [52].

DF can enhance the conversion of cholesterol into bile acids by altering the proportion of various bile acids metabolized in the liver [20,53]. Additionally, DF increases conjugated and primary bile acid levels, leading to significant changes in the bile acid-farnesoid X receptor signaling pathway. This regulation affects both bile acids and fatty acids [54]. Research has shown that foods rich in DF can lead to higher propionate levels, significantly inhibiting cholesterol synthesis [55]. Furthermore, the intake of PFB has been demonstrated to lower blood lipid levels through several mechanisms, including reducing cholesterol absorption and promoting bile acid secretion. Clinical trials have shown that adding PFP to the diet noticeably reduces plasma triglycerides, LDL-cholesterol, and total cholesterol levels in patients with dyslipidemia. It also tends to increase high-density lipoprotein cholesterol, which is known as “good cholesterol” [35]. This makes PFP a good choice for preventing coronary heart disease. Studies exploring the potential mechanisms behind DF’s cholesterol-lowering effects suggest that DF can block the enterohepatic circulation of bile acids by capturing them and thereby reducing cholesterol levels. When DF binds bile acids, they are absorbed and eventually excreted from the body. This loss of bile acids prompts the liver to convert more cholesterol into bile acids to compensate for the deficiency [56]. For instance, animal studies have demonstrated that the intake of PFS-IDF reduces serum triglyceride levels from 1.70 mmol/L to 1.00 mmol/L and serum total cholesterol levels from 4.61 mmol/L to 3.70 mmol/L in hamsters, with no significant effect on high-density lipoprotein levels. Additionally, consuming PFS-IDF significantly increases hamster feces’ total lipids, cholesterol, and bile acids levels by 120%, 127%, and 183%, respectively. Therefore, the reduced serum cholesterol observed in hamsters may be attributed to the high cation exchange capacity (42.5 equivalent/kg mequiv/kg) of PFS-IDF. This property allows it to disrupt, capture, and decompose mixed micelles of lipids, hindering lipid and bile acid absorption while enhancing the excretion of bile acids [39].

Furthermore, PFS has been found to contain resveratrol and pterostilbene, which can lower triglyceride and cholesterol levels in rat serum. Research indicates that the intake of PFS extract reduced cholesterol levels from 478.4 mg/dL to 278.5 mg/dL and triglyceride levels from 126.6 mg/dL to 83.2 mg/dL in rats on a high-fat diet. Additionally, the consumption of PFS improved platelet aggregation in vitro, showing a reduction of 16.06% when exposed to 64 μM adenosine diphosphate. It also enhanced cardiac function and facilitated acetylcholine-mediated aortic ring relaxation in rats fed a high-fat diet while alleviating vascular and liver abnormalities associated with such diets, as evidenced by liver sections showing reduced fibrosis and tubular reactions [38].

### 3.2. Hypoglycemia

Diabetes mellitus is the fourth leading cause of death worldwide, affecting approximately 3% of the global population [20]. According to the IDF, the prevalence of type 2 diabetes is projected to rise from 463 million cases in 2019 to 700 million by 2043 [57]. Plant-based DF is one of the most effective preventive measures against type 2 diabetes [58]. Research conducted by Lindström et al. (2006) [59] indicates that individuals with the highest DF intake experienced a 62% reduction in the progression from prediabetes to diabetes over 4.1 years.

The primary way that DF reduces blood glucose levels is through the properties of SDF and IDF. SDF has a high viscosity and remarkable water-holding capacity, while IDF can create a loose, porous network structure. This combination allows for the formation of a gel that delays gastric emptying and thickens the contents of the small intestine. As a result, this reduces the diffusion of nutrients and minimizes their contact with digestive enzymes [14]. PFBs are an excellent source of DF and serve as an ideal raw material for lowering blood glucose levels. For instance, Corrêa et al. (2014) [41] experimented using PFP-DF as the sole fiber source for rats. They found passion fruit DF could absorb water to form a viscous gel, clear the intestines, reduce triglycerides and LDL-cholesterol, and lower insulin and leptin levels, effectively reducing blood glucose. Additionally, Chau et al. (2004) [40] studied the hypoglycemic effects of PFS-IDF. They discovered that its cation exchange capacity was significantly higher than cellulose’s, even though both had similar water and oil-holding capacities. This enhanced ability allowed PFS-IDF to absorb glucose more effectively and inhibit amylase activity.

DF fermentation in the gut produces SCFAs, which can stimulate an increase in anorectic hormones while decreasing circulating ghrelin-releasing peptides [60]. Research conducted by Psichas et al. (2015) [61] confirmed that treating with 0.4 mmol of propionate for 15 min elevated plasma levels of glucagon-like peptide-1 from 8.7 pmol/L to 13.8 pmol/L and peptide YY from 53.8 to 76.5 pmol/L. The increase in glucagon-like peptide-1 and peptide YY helps suppress appetite and control blood glucose levels. Additionally, Salgado et al. (2010) [20] demonstrated that PFP can enhance blood glucose conversion to hepatic glycogen. In experiments where 5% PFP was added to the diets of diabetic rats, a significant reduction of 59% in blood glucose levels was observed, bringing them down to 112.6 mg/dL. The hepatic glycogen content in these diabetic rats was nearly equivalent to that of normal rats. This effect may result from PFP reducing carbohydrate digestion and absorption while increasing insulin sensitivity in adipose tissue. PFB also exhibits other anti-glycemic mechanisms, stemming from its high content of bound polyphenols in PFP-DF and betulin in PFS, which imparts significant antioxidant activity. Studies have shown that consuming 10 mg of betulin from PFS daily over a week increases average fat burning at rest by 39.5% compared with a placebo [25]. Furthermore, polyphenols have been found to lower blood sugar levels by inhibiting the activity of enzymes involved in glucose release, such as α-glucosidase and α-amylase. This inhibition varies based on the source and composition of the phenols [7]. Research by Li et al. (2019) [42] established that PFP polyphenols have a strong inhibitory effect on both α-glucosidase and α-amylase, demonstrating effective in vitro hypoglycemic activity.

### 3.3. Modulating Intestinal Flora

The gastrointestinal system is the most significant barrier tissue in the human body [62]. It not only promotes the digestion of food but also plays a crucial role in the mucosal immune system. However, an imbalance in intestinal microorganisms can compromise the intestinal barrier, resulting in various diseases such as diabetes, obesity, and several gastrointestinal disorders.

**Figure 2:**
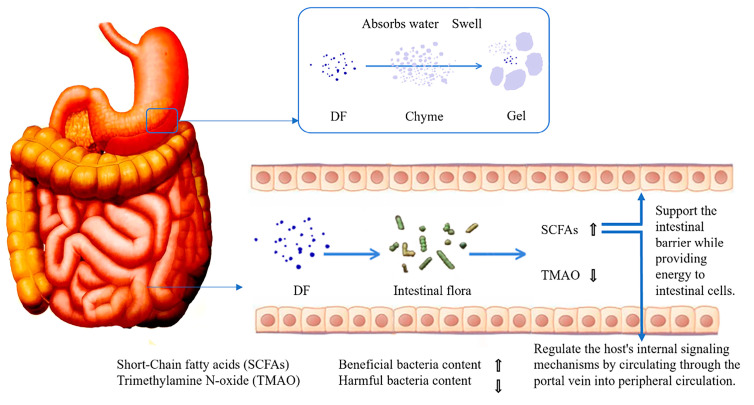
The process of DF regulating intestinal microbiota.

DF with prebiotic properties exerts gut-protective effects through multiple synergistic mechanisms [63]. First, DF absorbs water to form viscous gels through swelling, a physical characteristic that not only delays gastric emptying but also encapsulates chyme, thereby effectively reducing direct contact between pathogens and intestinal epithelial tissues. Second, DF modulates gut microbiota composition by significantly increasing the abundance of beneficial bacteria. This action produces dual metabolic effects. Elevated concentrations of SCFAs, which serve as rapid energy substrates for colonocytes while also entering systemic circulation via the portal vein to regulate host endocrine functions, notably enhancing the secretion of peptide YY and glucagon-like peptide-1 peptides with acute appetite-suppressing effects and a marked reduction in trimethylamine N-oxide levels, a metabolite strongly associated with increased risks of metabolic disorders including diabetes mellitus, atherosclerosis and [64]. These intricate regulatory mechanisms are schematically illustrated in Figure 2.

The IDF can reduce the contact between carcinogens and the inner surface of the intestinal mucosa by diluting fecal toxins and shortening transit time. Additionally, it can decrease inflammatory responses by lowering blood cholesterol levels and reducing the concentration of secondary bile acids in the colon [65]. Research has shown that butyrate can inhibit the expression of pro-inflammatory cytokines by blocking the activation of nuclear factor κB, thereby mitigating inflammation [66]. Furthermore, high propionate concentrations promote the expression of mucin genes, specifically Mucin2 and Mucin4, in the intestinal mucus layer, which helps protect the intestinal barrier [63]. PFP-DF contains a significant amount of pectin, making it an excellent substrate for fermentation by intestinal bacteria. PFP-SDF can be fermented by gut flora to produce SCFAs, which enrich the populations of beneficial bacteria such as *Lactobacillus* and *Bifidobacterium* while inhibiting pathogenic bacteria [43]. Additionally, it has been reported that PFP can act as a carbon source for microbial fermentation, leading to increased concentrations of SCFAs in feces. Among these acids, acetate has shown anti-inflammatory activity in in vitro models of experimental colitis in mice, while butyrate provides significant protective effects on the mucosal barrier [45].

In an experiment conducted by Casarotti et al. (2020) [44] on the effects of passion fruit fermented goat milk on colonic microbiota, it was found that PFP-DF promoted the growth of beneficial bacteria such as *Bifidobacterium* and *Lactobacillus* in the colon. This increase in beneficial bacteria was attributed to several factors, including the optimal pH level provided by PFP-DF, which is conducive to *Lactobacillus* growth, as well as protein hydrolysis during the fermentation of goat milk, which further enriched these bacteria. Additionally, PFP-DF reduced the abundance of potentially harmful bacteria such as *Prevotella*, *Megamonas*, *Bacteroides*, and *Succinivibrio* in a simulated colon created from the feces of obese patients. These bacteria are typically found at higher levels in the feces of obese individuals and are associated with producing SCFAs. The consumption of PFP-DF fermented goat milk helped to balance the microbial composition in the colon. Furthermore, a study by Abboud et al. (2019) [46] revealed that the intake of 10 mg/kg of PFP-SDF resulted in a 50.33% reduction in glutathione consumption, a 31.00% reduction in gastric mucosal fluid, and an 87.17% decrease in ethanol-induced gastric ulcer lesions in rats. This suggests that PFP-SDF effectively reduces gastric ulcer lesions in rats by minimizing the consumption of glutathione and gastric mucosal fluid.

### 3.4. Slimming

Obesity is a significant risk factor for several diseases, including cancer, cardiovascular diseases, and type 2 diabetes. Statistics indicate that between 1975 and 2014, the global obesity rate (Body mass index ≥ 30 kg/m^2^) increased significantly: for men, it rose from 3.2% to 10.8%, and for women, from 6.4% to 14.9% [67]. Anderson et al. (2008) [58] conducted cross-sectional and prospective cohort studies to examine the relationship between DF intake and obesity. The findings revealed a significant negative correlation between DF intake and the risk of obesity. Specifically, individuals with the highest DF intake had a risk of obesity that was approximately 30% lower than those with the lowest DF intake [58].

The high viscosity of SDF can delay the diffusion of nutrients within intestinal chyme and slow the gastric emptying rate. DF can trap cholesterol and other substances through its network structure, leading to increased bile acids and cholesterol excretion, which helps remove fat deposits [68]. Additionally, DF can regulate various gastrointestinal hormones, such as incretin, orexin, and agouti-related peptides [60]. Research conducted by Lucas et al. (2022) [47] suggests that the intake of particular fiber forms, such as PFP or PFS, can prevent fat accumulation in the body and protect against liver damage. Furthermore, the energy density of a diet is an important factor influencing obesity, as it relates to the content of fat, DF, and water. Studies indicate that participants in a high-fat/low-fiber group lost an average of 0.7 kg (95% CI 0.7–1.7 ± 0.1 kg) over three years, whereas those in a low-fat/high-fiber group lost an average of 3.1 kg (95% CI 2.3–3.9 kg) [59]. Animal research has shown that obesity accumulation is also linked to increased oxidative stress and inflammatory responses. PFP, rich in DF and polyphenols, can decrease lipid peroxidation in the liver and adipose tissue while reducing inflammatory cytokines in the blood. Studies have demonstrated that the intake of PFP diminishes the activation of c-Jun N-terminal kinase and inhibitor of kappa B kinase α/β kinase, enhances the antioxidant defense capacity of rat liver and epididymal adipose tissue, improves inflammatory states, reduces body fat, and counteracts weight gain [48]. In the human clinical trial conducted by Kitada et al. (2017) [49], piceatannol extracted from PFS demonstrated significant beneficial effects in obese male participants, manifested as reduced blood pressure and heart rate, along with improved insulin sensitivity. However, it is noteworthy that these effects were not observed in non-overweight male participants, overweight female participants, or non-overweight female participants. The precise mechanisms underlying these population-specific differences remain unclear and warrant further investigation.

## 4. Application of Passion Fruit DF in Food

As shown in Table 3, PFB has diverse applications in the food industry. In dairy products, adding PFB can improve the taste of yogurt, enhance its nutritional value, and extend its shelf life. In baked goods, PFB can help compensate for the nutritional deficiencies of gluten-free products and positively affect the quality and characteristics of items such as biscuits and bread. In meat products, PFB can enrich nutrients, reduce fat and cholesterol content, and improve the quality of products such as sausages, surimi, and hamburgers. These applications offer new opportunities for the food industry to develop high-nutritional-value products.

### 4.1. Application in Dairy Products

Yogurt is a healthy food created through the fermentation of milk. Incorporating PFB into a yogurt recipe can enhance its flavor, boost its nutritional content, prolong its shelf life, and increase consumer approval.

Adding PFP to fermented milk enhances its nutritional value and the stability of lipids in milk beverages. Research shows that the esterification degree of PFP pectin exceeds 70%, enabling it to form gels quickly at high temperatures, positively impacting lipid distribution [82]. Espírito-Santo et al. (2013) [69] conducted a study in which they added PFP to milk for fermentation. The results indicated that the DF in PFP provided a network structure for the yogurt curd, allowing extracellular polysaccharides to adhere and become embedded within it. As a result, whey separation in the yogurt was significantly reduced during storage, and the overall structure became more stable. Although adding PFP slightly decreased the sensory evaluation scores, the unique physiological benefits provided by the DF from passion fruit led to increased acceptability of the fermented milk [70]. In another study, do Espírito Santo et al. (2012) [70] used whole milk and skim milk as substrates, along with four different probiotics (two strains of *Lactobacillus acidophilus* and two strains of *Bifidobacterium lactis* subsp.) as fermenting agents to prepare yogurt with PFP included. The introduction of PFP reduced the milk’s maximum acidification rate and shortened the skim milk’s fermentation time.

Salgado et al. (2021) [53] researched the effects of adding PFP-DF to donkey milk. They developed five types of yogurt for comparison: donkey milk yogurt, donkey milk inulin yogurt, donkey milk apple peel DF yogurt, donkey milk PFP-DF yogurt, and traditional milk yogurt. The results indicated that PFP-DF yielded donkey milk yogurt with a viscosity most similar to milk yogurt. While adding PFP-DF improved the yogurt’s nutritional value, it negatively impacted the sensory score, affecting acceptability. Therefore, optimizing the formulation and enhancing its nutritional value is essential to improving overall acceptability. In another study, Santos et al. (2017) [83] compared fermented and unfermented PFP pectin beverages with commercial pectin beverages. Their findings showed that PFP pectin improved the survival rate of *Lactobacillus* rhamnosus in both fermented and non-fermented beverages under simulated gastrointestinal conditions. This suggests that PFP-derived pectin is more suitable than standard commercial pectin as an additive in probiotic fermented foods. Additionally, PFS is effective for developing dairy beverages. Rotta et al. (2020) [71] extracted polyphenols from PFS to prepare dairy beverages. Their experiments demonstrated that the inclusion of PFS polyphenols not only enhanced the antioxidant activity of dairy beverages and reduced lipid oxidation during sterilization and storage.

PFB can also be used in ice cream, cheese, and various dairy beverages to create new products with high nutritional value. For instance, de Oliveira et al. (2022) [72] utilized passion fruit and PFP pectin to produce lactose-free ice cream. These natural additives enhanced the ice cream’s aroma and added a significant amount of phenolic compounds, vitamins, and other beneficial substances. Additionally, Costa et al. (2020) [73] incorporated PFP into Brazilian cheese, and their results demonstrated that PFP inhibited important pathogenic bacteria such as *Listeria* and *Staphylococcus aureus*, while lactic acid bacteria were unaffected.

### 4.2. Application in Noodle Products

Noodles are a popular staple food worldwide, but many patients with celiac disease are allergic to gluten-containing products. Therefore, developing and researching gluten-free products is essential [84].

Adding PFP can address the nutritional deficiencies commonly found in gluten-free products. Ribeiro et al. (2018) [74] studied gluten-free pasta made with rice and corn flour, comparing it to pasta with PFP. The results indicated that adding PFP increased the cooking time of the pasta, improved the retention of soluble solids, and enhanced its color. When 10% PFP was added, consumer acceptance reached 70%; however, higher levels of PFP tended to decrease acceptance. Researchers conducted additional experiments to improve the acceptability of noodles made with passion fruit DF. In these tests, the TDF content of the PFP used was 75.3%, with SDF at 20.6%. While the presence of PFP contributed to the gelatinization and retrogradation of wheat flour, the competition for water between PFP and wheat flour hindered the formation of the gluten network, negatively affecting the cooking characteristics. Ultimately, selecting a 6% PFP concentration enhanced both the dry noodles’ functional characteristics and nutritional value while preserving their cooking qualities, chewiness, and overall sensory attributes [75].

In addition to noodles, the development of DF biscuits is gaining popularity. DF has a positive impact on the shelf life of biscuits. Adding high levels of PFP (pectin) enhances the nutritional value of the biscuits. It inhibits the growth of bacteria such as *Escherichia coli*, *Staphylococcus aureus*, and *Salmonella*, extending the product’s storage period. While there is no significant difference in sensory evaluation, consumer acceptance has increased [76]. Research has also been conducted on the application of DF in bread. For instance, some researchers have used PFP pectin as a bread improver. Compared with bread enhanced with soybean dregs or without any improver, the dough treated with PFP pectin exhibited greater tensility, elasticity, viscosity, and cohesiveness. Consequently, the overall quality of the dough was superior to that of the other two types of bread [77].

### 4.3. Application in Meat Products

Meat products provide various nutrients, including amino acids, proteins, fats, vitamins A and B, and essential minerals. However, they also contain high levels of saturated fats and cholesterol. The addition of DF not only enhances the nutrient profile and improves the cooking quality of meat products, but it also helps to reduce their fat and cholesterol content [85]. Sausages, which are a common type of meat product, particularly benefit from the inclusion of PFP. Adding PFP to sausages decreases weight loss during the smoking process and enhances microbial stability over 90 days, likely because of its potential bactericidal properties. Moreover, PFP’s antioxidant abilities significantly reduce lipid oxidation in lamb sausages [78]. Additionally, research conducted by Hang et al. (2022) [79] on tilapia fish paste demonstrated that incorporating PFP alters the water dynamics of the fish paste gel. Specifically, a 4% addition of PFP resulted in increased bound water, decreased free water, improved hardness, enhanced gel strength, and greater water-holding capacity while reducing cooking loss. These changes contribute to a higher quality of tilapia fish paste gel.

Using DF to develop new types of hamburger meat products is a promising choice in the fast-food industry. Adding 2.5% PFP to hamburgers improves cooking yield, water retention, and fat retention and inhibits the growth of aerobic thermophilic bacteria and intestinal bacteria, enhancing product acceptability [80]. Filho et al. (2021) [86] used mature yellow PFP and green banana powder as additives to create hamburgers. The results indicated that green banana powder improved the hamburgers’ appearance, aroma, flavor, texture, and shrinkage rate. Meanwhile, PFP not only increased the elasticity and reduced the moisture content but also boosted the mineral content of the hamburgers. However, due to the reduced biomass, the pH of the hamburger reached 6.4, which is near the threshold for spoiled meat. Despite this, using PFP to develop new meat products presents an opportunity to reduce industrial waste, and this research direction holds significant exploratory value.

There is limited research on using PFS as an additive in meat product development, both domestically and internationally. However, experiments have shown that appropriately supplementing PFS oil during broiler rearing can positively impact meat quality. For instance, Zanetti et al. (2021) [81] found that adding PFS oil to broiler diets resulted in softer skin and decreased susceptibility to oxidative damage, attributed to the antioxidant properties of the seed oil. In another experiment by Assunção et al. (2023) [87], broilers were given a diet supplemented with 0.9% PFS oil and subjected to 36 days of daily 8 h heat stress. The results indicated that adding PFS oil enhanced the heat resistance of proteins in chicken breast meat, suggesting its potential to mitigate oxidative stress and inflammatory responses. This finding has significant implications for further research.

## 5. Conclusions and Prospect

PFB is a novel health food ingredient with significant potential, containing active substances such as DF and polyphenols. These components play vital roles in human health, including reducing blood lipids and glucose levels, regulating gut microbiota, and supporting weight management. The DF in PFB is characterized by high pectin content and bound polyphenols, which contribute to its structural complexity and multifunctionality. Although recent studies have emphasized the prospects of PFB, key limitations still exist. For instance, the bioavailability of polyphenols in PFB remains poorly understood. Furthermore, the colonic-targeted release kinetics of DF-polyphenol complexes (referring to the site-specific release patterns of bioactive compounds in the colon) remain unquantified, as current in vitro simulation systems fail to accurately reproduce the synergistic effects between intestinal pH gradients and microbial enzymatic activity. This limitation impedes a mechanistic understanding of dietary fiber and polyphenol release. To address current research challenges, future studies should employ modern nutritional science and metabolomics technologies to systematically evaluate the integrated effects of PFB on gut microbiota and metabolic health. As exemplified by the work of Cao et al. (2021) [88], who utilized high-performance liquid chromatography–tandem mass spectrometry analysis (quasi-targeted metabolomics) to comprehensively analyze the impacts of simulated gastrointestinal digestion on the bioactive components, bioactivity, and bioaccessibility of ethanol extracts from PFP. This research paradigm provides a valuable reference for the in-depth development of PFB.

Additionally, current research still faces technical barriers; conventional extraction methodologies for DF and polyphenols (acid-based extraction, alkaline-based extraction) demonstrate high energy consumption, substantial solvent residues, and suboptimal recovery rates of bioactive components. Simultaneously, the lack of dynamic models analyzing how Passiflora edulis maturity stages influence bioactive components leaves critical uncertainties about developmental variations in DF and bound polyphenol composition.

In product development phases, while PFB incorporation enhances nutritional profiles and textural properties, it paradoxically adversely affects sensory evaluations. In gluten-free pasta products, PFB-derived DF compensates for nutritional deficiencies but compromises cooking characteristics. Furthermore, excessive DF utilization may impede nutrient bioavailability and induce adverse gastrointestinal responses, including abdominal distension and diarrhea. Similarly, polyphenol overconsumption risks disrupting microbial equilibrium, potentially exacerbating iron deficiency-induced anemia through interference with iron homeostasis. Therefore, well-designed clinical trials are urgently required to establish the optimal incorporation levels and functional properties of PFB in various food matrices, thereby providing scientific dietary guidance for consumers. Notably, PFP has emerged as a high-quality raw material for value-added bioproducts, demonstrating significant potential in circular economic development. As evidenced by Infante et al. (2024) [89], bioconversion of PFP (rich in cellulose and hemicellulose) can efficiently produce xylitol (yielding up to 14.79 g/L). This finding establishes a novel approach for valorizing agricultural by-products. Such research not only aligns with sustainable development principles but also opens new avenues for the comprehensive utilization of PFB.

To address these challenges, future investigations should leverage modern nutritional science and metabolomics technologies to systematically evaluate PFB’s comprehensive impact on gut microbiota and metabolic health. For instance, metabolomic profiling could elucidate intestinal metabolic pathways of DF and polyphenols and their microbiota-modulating effects. Furthermore, clinical trials are warranted to establish optimal incorporation levels and specific functionalities of PFB in novel food matrices, enabling targeted nutritional recommendations for consumers. As a functional food ingredient, PFB demonstrates significant potential. Nevertheless, achieving industrial implementation necessitates resolving critical issues spanning extraction optimization, mechanistic clarification, and application challenges. Through the integration of advanced technological approaches and rigorous fundamental research, the development of safer, more efficient PFB-derived products appears feasible. Such advancements promise dual benefits: delivering superior health-oriented options to consumers while enhancing agricultural profitability and facilitating industrial upgrading.

## Figures and Tables

**Table 1:** DF and active ingredients of passion fruit.

Color	Research Object	TDF (SDF/IDF)	Phenolics	Other Active Ingredients	2,2-Diphenyl-1-picrylhydrazyl (DPPH)	Allion Bluetooth Test Suite (ABTS)	Function Characteristics	Reference
yellow	Brazil-PFP	61.16 ± 1.02% (Pectin: 37.67 ± 0.97%)	1061.87 ± 25.00 mg/100 g	Quercetin: 760.00 ± 32.07 mg/100 g	1.69 ± 0.03 g/100 mL	2.22 ± 0.01 g/100 mL	—	[19]
	Brazil-PFP	(3.49%/54.27%)	4.2 mg/g	—	—	—	—	[20]
	Colombia-PFP	63.40 ± 0.10%	64.94 ± 0.27 mg Gallic acid equivalents (GAE)/g	—	—	—	Water holding capacity (WHC): 11.48 ± 2.54 mL/g Oil holding capacity (OHC): 5.07 ± 0.63 mL/g Swelling rate (SR): 14.72 ± 2.22 mL/g	[21]
	Colombia-PFP	—	30.19 ± 3.00 mg GAE/g	—	10.56 ± 0.80 μg/mL	—	—	[22]
	Colombia-PFP	—	8.34 ± 0.83 mg GAE/g	—	718.91 ± 40.55 μg/mL	—	—	[22]
	Mexico-PFP	57.93 ± 2.72% (11.75 ± 1.21%/46.18 ± 1.21%)	482.56 mg GAE/100 g	—	—	—	—	[23]
	Ecuador-PFP	—	24.96 ± 2.00 mg GAE/g	—	32.93 ± 2.88 μg/mL	—	—	[22]
	Côte d’Ivoire-PFP	81.9 ± 1.40% (17.90 ± 0.40%/62.40 ± 0.70%)	—	—	—	—	WHC: 4.10 ± 0.10 g/g OHC: 5.20 ± 0.1 g/g	[24]
	Brazil-PFS	65.60 ± 0.52%	346.69 ± 6.58 mg/100 g	Anthocyanin: 4598.70 ± 119.73 μg/100 g Kaempferol: 375.32 ± 13.50 mg/100 g	1.18 ± 0.03 g/100 mL	3.84 ± 0.08 g/100 mL	—	[19]
	Brazil-PFS	65.60 ± 0.52%	—	—	—	—	—	[25]
	India-PFS	55.8 ± 2.1 (3.6 ± 0.6/52.2 ± 1.1)%	—	—	—	—	WHC: 2.9 ± 0.06 g/g OHC: 4.1 ± 0.05 g/g SR: 15.7 ± 0.08 mL/g	[26]
purple	Brazil-PFP	61.68 ± 1.31% (Pectin: 32.85 ± 1.20%)	1570.80 ± 26.76 mg/100 g	Anthocyanin: 103686.48 ± 542.11 μg/100 g Kaempferol: 74.70 ± 1.44 mg/100 g	6.98 ± 0.20 g/100 mL	9.37 ± 0.05 g/100 mL	—	[19]
	Colombia-PFP	—	5.08 ± 0.48 mg GAE/g	—	298.57 ± 18.31 μg/mL	—	—	[22]
	Brazil-PFS	55.06 ± 0.35%	325.69 ± 1.18 mg/100 g	Anthocyanin: 8232.41 ± 6.54 μg/100 g	6.30 ± 0.08 g/100 mL	4.76 ± 0.03 g/100 mL	—	[19]
	Brazil-PFS	55.06 ± 0.35%	—	—	—	—	—	[25]
orange	Brazil-PFP	62.14 ± 2.62% (Pectin: 21.55 ± 0.55%)	2584.91 ± 96.67 mg/100 g	Quercetin: 800.13 ± 24.18 mg/100 g Kaempferol: 229 ± 8.90 mg/100 g	2.45 ± 0.03 g/100 mL	2.95 ± 0.02 g/100 mL	—	[19]
	Brazil-PFS	51.47 ± 0.60%	429.33 ± 0.19 mg/100 g	Anthocyanin: 293.36 ± 6.75 μg/100 g Quercetin: 120.41 ± 2.82 mg/100 g	2.68 ± 0.03 g/100 mL	3.87 ± 0.00 g/100 mL	—	[19]

**Table 2:** Physiological functions of PFB.

Physiological Function	Research Object	Research Object	Potential Prevention Mechanisms	References
Lowers Blood Lipids	PFP	Human clinical trial, Animal test	The levels of triglycerides, LDL-cholesterol, and total cholesterol decreased, while the level of high-density lipoprotein cholesterol increased.	[35,36,37]
	PFS-Ethanol extract	Animal test	Reduce the levels of triglycerides and cholesterol in rat serum.	[38]
	PFS-IDF	Animal test	Impede enterohepatic circulation; enhance bile acid excretion.	[39]
Hypoglycemia	PFS-IDF	In vitro testing	Absorption of glucose and inhibition of amylase activity.	[40]
	PFP-DF	Animal test	Reduce triglycerides and LDL-cholesterol, and reduce insulin or leptin levels.	[41]
	PFP	Animal test	Stimulate hepatic glycogen synthesis; enhance insulin sensitivity in adipose tissue.	[20]
	PFP-Polyphenol	In vitro testing	Inhibition of α-Glucosidase and α-Amylase Activities.	[42]
Modulating Intestinal Flora	PFP-SDF	Animal test	Enrich beneficial bacteria and inhibit pathogenic bacteria.	[43]
	PFB (PFP, PFS, Pomace) goat milk yogurt	In vitro testing		[44]
	PFP	Animal test, In vitro testing	Enhance the content of short-chain fatty acids (SCFAs), exhibiting anti-inflammatory activity.	[45]
	PFP-SDF	Animal test	Reduced ethanol-induced gastric ulcer lesions.	[46]
Slimming	PFP, PFS	Animal test	Prevent accumulation of body fat and liver damage.	[47]
	PFP	Animal test	Improve the antioxidant defense capability of rat liver and epididymal adipose tissue; improve the inflammatory state and reduce body fat.	[48]
	PFS	Human clinical trial	significantly improved blood pressure and heart rate while enhancing insulin sensitivity in obese male subjects	[49]

**Table 3:** Application of PFB in food.

	Product Form	Additives	Advantages	Disadvantages	Conclusions	Reference
Dairy Products	Milk yogurt	PFP	Shorten fermentation time, improve textural properties, increase the content of lactic acid bacteria, and enhance nutritional value.	Sensory evaluation has been marginally decreased	The yogurt with a 1% PFP addition exhibited the optimal characteristics.	[69]
	Milk yogurt	PFP			Skim milk is more suitable than whole milk for the development of PFP yogurt. Physiologically active modifiers	[70]
	Donkey milk yogurt	PFP-DF Apple peel-DF Inulin	Improved curd quality and enhanced nutritional value	Sensory evaluation has been marginally decreased	PFP-DF is more suitable for the development of DF yogurt compared with apple peel-DF and inulin.	[53]
	Milk-based compound beverage	PFS-polyphenol	Increase antioxidant activity and reduce lipid peroxidation.	—	PFS-polyphenol extract can prevent lipid oxidation in dairy beverages during storage and digestion.	[71]
	Lactose-free ice cream	Passion fruit pulp and PFP-pectin	Improved odor and enhanced nutritional value	—	The addition of whole passion fruit can develop products with lower processing degrees, no food additives, and higher micronutrient content.	[72]
	cheese	PFP	Inhibition of harmful bacteria growth, enhancement of nutritional value	—	PFP inhibits the growth of harmful bacteria while having no significant effect on lactic acid bacteria.	[73]
Noodle Products	noodle	PFP Rice flour corn flour	Improve nutritional value	Damage to cooking characteristics	PFP is more suitable for the development of noodles than a blend of rice flour and corn flour	[74]
	noodle	PFP	Improve nutritional value	Damage to cooking characteristics	Additives with an addition amount of 6% exhibited the optimal characteristics for noodles.	[75]
	Cookie	PFP	Inhibition of harmful microbial growth, extension of product shelf life.	—	The biscuits with an additive content of 30% exhibited the optimal characteristics.	[76]
	Bread	PFP-pectin Okara	Improved texture characteristics, enhanced nutritional value	—	PFP-pectin is more suitable for the development of bread compared with soybean dregs.	[77]
Meat Products	Sausage	PFP	Reduce smoking loss, inhibit the growth of harmful bacteria, reduce lipid oxidation, and enhance nutritional value.	Sensory evaluation has been marginally decreased	The sausage with an additive content of 6% exhibited the optimal characteristics.	[78]
	Surimi	PFP	Improved texture characteristics, reduced cooking loss rate, and enhanced nutritional value.	—	Additives with an addition level of 4% exhibited the optimal properties in surimi.	[79]
	Pork burger	PFP	Improved texture characteristics, enhanced cooking yield, inhibition of harmful bacteria growth, inhibition of intestinal bacteria growth, and enhanced nutritional value.	The pH of hamburger meat is critical at the edge of spoilage.	The burger with an additive concentration of 2.5% exhibited the optimal characteristics.	[80]
		PFP Green banana			PFP is more suitable than green bananas for the development of hamburger meat.	[81]

## Data Availability

No new data were created or analyzed in this study. Data sharing is not applicable to this article.

## Abbreviations

The following abbreviations are used in this manuscript:

PFB	Passion Fruit By-products
PFP	Passion Fruit Peel
PFS	Passion Fruit Seeds
DF	Dietary Fiber
LDL	Low-Density Lipoprotein
SDF	Soluble Dietary Fiber
IDF	Insoluble Dietary Fiber
TDF	Total Dietary Fiber
DPPH	2,2-Diphenyl-1-Picrylhydrazyl
ABTS	Allion Bluetooth Test Suite
GAE	Gallic Acid Equivalents
WHC	Water Holding Capacity
OHC	Oil Holding Capacity
SR	Swelling Rate
PLE	Pressurized Liquid Extraction
UAE	Ultrasound-Assisted Extraction
MAE	Microwave-Assisted Extraction
ORAC	Oxygen Radical Absorbance Capacity
TE	Trolox Equivalent
SCFAs	Short-Chain Fatty Acids
